# The Role of Operating Temperature on Pore‐Scale Gas Transport in Polymer Electrolyte Membrane Electrolyzers

**DOI:** 10.1002/advs.202507606

**Published:** 2025-09-08

**Authors:** Chaeyoung T. Ham, Pranay Shrestha, Leya Kober, Sergey Gasilov, M. Adam Webb, Aimy Bazylak

**Affiliations:** ^1^ Bazylak Group Department of Mechanical & Industrial Engineering University of Toronto 5 King's College Road Toronto ON M5S 3G8 Canada; ^2^ Canadian Light Source 44 Innovation Boulevard Saskatoon SK S7N 2V3 Canada

**Keywords:** 3D bubble distributions, bubble transport, hydrogen, operando X‐ray computed tomography, operating temperature, polymer electrolyte membrane electrolyzer, two‐phase flow

## Abstract

In this study, the effects of operating temperature on pore‐scale gas bubble transport in a carbon‐based anode porous transport layer (PTL) of a polymer electrolyte membrane (PEM) electrolyzer is revealed using operando X‐ray computed tomography (CT). Higher temperature operation (80 °C compared to 40 °C) led to a lower total gas bubble volume fraction in the PTL (0.25 to 0.17; 32% lower), which is attributed to improved water transport and gas removal. Higher temperatures led to fewer bubbles in the PTL (13% less at 80 °C compared to 40 °C) as well as a greater occurrence of smaller bubbles (35% increase in skewness of the bubble size distribution), and this behavior is attributed to faster rates of nucleation and bubble detachment from the electrode. The formation of gas slugs is also observed in the anode channels at 60 °C and 80 °C, which is attributed to the faster detachment and coalescence of bubbles from the PTL/flow field interface.

## Graphical abstract

This study clarifies the critical relationship between operating temperature and two‐phase flow behavior in polymer electrolyte membrane (PEM) electrolyzers by investigating pore‐scale gas bubble transport in the anode porous transport layer. Higher temperatures (80 °C vs 40 °C) result in a lower total bubble volume fraction and smaller bubble diameters, driven by enhanced water transport and increased bubble detachment rates.

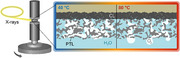

## 1 Introduction

Effective energy storage solutions are crucial for addressing the intermittency and fluctuating supply of wind and solar power,^[^ ^1^, ^2^ ^]^ and integrating these renewable sources supports the development of a more sustainable energy infrastructure.^[^ ^3^, ^4^ ^]^ To tackle challenges in energy storage, hydrogen has recently been regarded as a promising energy storage vector, as it can be utilized in fuel cells to provide on‐demand, emission‐free electricity for various applications including automotive,^[^ ^5^ ^]^ aviation,^[^ ^6^ ^]^ and stationary power systems,^[^ ^7^ ^]^ thereby reducing the reliance on fossil fuels in the current global energy mix.^[^ ^8^, ^9^, ^10^ ^]^ A key method of hydrogen production is via polymer electrolyte membrane (PEM) water electrolysis, where electricity is used to split water into hydrogen and oxygen.^[^ ^10^, ^11^, ^12^ ^]^ When powered by renewable sources, this process can be free of greenhouse gas emissions, enabling a complete carbon‐neutral cycle from electricity generation to use. Despite this advantage, the widespread adoption of PEM electrolyzers remains limited by high costs and performance inefficiencies, which are tied to both suboptimal material design and insufficient understanding of interfacial transport phenomena.^[^ ^13^, ^14^, ^15^ ^]^ To lower hydrogen costs and achieve commercialization, the U.S. Department of Energy has set cell performance targets of 1.8 V at 3.0 A cm^−2^ and a hydrogen production cost of $2/kg H_2_ by 2026.^[^ ^16^ ^]^

A critical challenge in PEM electrolyzer operation is inefficient two‐phase flow of liquid water and gaseous oxygen at the anode.^[^ ^17^, ^18^, ^19^, ^20^, ^21^ ^]^ At high current densities, oxygen bubbles produced at the anode accumulate in the anode porous transport layer (PTL) hindering the efficient transport of reactant liquid water to the catalyst or physically obstructing reaction sites.^[^ ^22^, ^23^ ^]^ Improper oxygen bubble management can lead to mass transport limitations,^[^ ^24^ ^]^ non‐uniform current distributions,^[^ ^25^ ^]^ and membrane degradation,^[^ ^22^ ^]^ all of which are detrimental to performance. To mitigate these issues, numerous studies have focused on understanding the influence of current density, flow rate, and PTL microstructure on two‐phase transport behavior.^[^ ^17^, ^19^, ^23^, ^26^, ^27^, ^28^ ^]^ These efforts have established that oxygen removal in the PTL is predominantly capillary‐driven and is heavily influenced both by the applied current and PTL microstructure.^[^ ^19^, ^28^, ^29^, ^30^, ^31^, ^32^, ^33^, ^34^ ^]^

One key operational parameter that has received comparatively limited attention is operating temperature. High‐temperature operation has been shown to enhance electrolyzer efficiency, improve ionic conductivity, and increase hydrogen production rates,^[^ ^35^, ^36^ ^]^ highlighting a key area for future PEM electrolyzer research. Importantly, temperature also affects fluid properties such as viscosity, surface tension, and gas solubility, which are all relevant to multiphase transport. Recent works have begun to explore temperature effects on the two‐phase flow in the anode flow field (FF) channels. Selamet et al.^[^ ^20^ ^]^ reported an increase in gas volume occupancy in the channels of a mesh‐type anode FF at higher temperatures (from 25 °C to 40 °C) due to higher water evaporation rate and lower gas density. Lee et al.^[^ ^37^ ^]^ found that while increasing the temperature (from 40 °C to 80 °C) led to higher gas content in the channels, a decrease in water viscosity led to more uniform water delivery to reaction sites, which ultimately resulted in lower mass transport losses. While these studies reveal important macroscopic trends in the channels, they do not directly address how temperature alters gas transport behavior within the PTL.

While a few modelling studies have specifically explored temperature effects on the two‐phase flow in the PTL,^[^ ^18^, ^30^, ^38^ ^]^ these have primarily relied on non‐standard cell architectures. For instance, Ojong et al.^[^ ^30^ ^]^ predicted via numerical modelling that decreasing the operating temperature would reduce the average bubble diameter at the PTL/catalyst‐layer interface, potentially mitigating catalyst bubble coverage and improving mass transport. Li et al.^[^ ^18^ ^]^ employed optical imaging and modelling to show that increasing the operating temperature (40 °C to 80 °C) increased both the bubble diameter and bubble growth velocity in the PTL. However, the model PTLs used in both studies by Ojong et al.^[^ ^30^ ^]^ and Li et al.^[^ ^18^ ^]^ consisted of straight‐through cylindrical pores, which diverge significantly from conventional PTL structures with stochastically distributed irregular pore morphologies, limiting the applicability of their results in understanding gas transport behavior in more representative PEM electrolyzer architectures.

To gain a better understanding of two‐phase flow behavior in the PTL, direct visualization of an operating PEM electrolyzer can be conducted. More recently, Lee et al.^[^ ^38^ ^]^ used operando neutron radiography to reveal that an increase in operating temperature (from 40 °C to 80 °C) resulted in lower gas saturation in the PTL and promoted more uniform gas distributions. While valuable, this work only captured spatially‐averaged two‐dimensional (2‐D) data and assumed a homogeneous porosity for the PTL, providing no pore‐scale insights such as bubble diameter. Given the structural heterogeneity and anisotropy of PTLs, a three‐dimensional (3‐D) characterization is essential to fully resolve the temperature‐dependent mechanisms influencing fluid transport in these PTLs. Notably, previous studies have reported seemingly conflicting temperature‐dependent effects on gas transport behavior in PEM electrolyzers, such as gas volume and bubble diameter,^[^ ^18^, ^30^, ^37^ ^]^ highlighting the need for direct 3‐D imaging to clarify these mechanisms.

Operando X‐ray computed tomography (CT) is a powerful tool for visualizing 3‐D dynamic interfacial phenomena at the pore‐scale with high spatial resolution.^[^ ^39^, ^40^, ^41^, ^42^, ^43^, ^44^, ^45^, ^46^, ^47^, ^48^, ^49^, ^50^ ^]^ Satjaritanun et al.^[^ ^51^ ^]^ used X‐ray CT to quantify the oxygen content within various regions of the PTL (using a carbon model PTL) at different flow rates, where they revealed that oxygen transport was governed by preferential pathways. De Angelis et al.^[^ ^52^ ^]^ successfully observed oxygen distributions in a Ti PTL using stained water and demonstrated that oxygen content increased with current density, and the oxygen saturation decreased from the catalyst layer to the channel. Following these studies, Kulkarni et al.^[^ ^53^ ^]^ examined the effect of an MPL on the oxygen transport through the PTL and found that the number of oxygen transport pathways increased with the addition of the MPL. While these studies investigated the impact of flow rate, current density, and material properties on gas transport in the PTL, an examination of the impact of operating temperature on gas transport using 3‐D imaging is still needed. Operando X‐ray CT presents a valuable opportunity to provide clear mechanistic insights on the temperature‐dependence of gas bubble transport in the PTL.

In this study, we employ operando synchrotron X‐ray CT to investigate the temperature‐dependent behavior of pore‐scale gas transport in PEM electrolyzers. To enable sufficient X‐ray transmission and resolution, symmetric carbon‐based PTLs are used as a model system. Carbon‐based PTLs offer favorable characteristics for operando X‐ray imaging due to its lower X‐ray attenuation whereas titanium PTLs, though more representative of commercial PEM electrolyzers, poses significant imaging challenges due to their higher density and X‐ray attenuation.^[^ ^51^ ^]^ While carbon PTLs have larger pore sizes and higher intrinsic hydrophobicity than titanium PTLs, which may lead to differences in absolute capillary behavior, the temperature‐sensitive parameters we investigate here which govern two‐phase transport mechanisms are common to both material systems. Thus, the use of carbon‐based PTLs allows us to isolate and study the fundamental effects of temperature on two‐phase transport in a stochastic porous medium, ensuring mechanistic relevance while leveraging the high spatial and temporal resolution afforded by synchrotron X‐ray CT.

**Figure 1:**
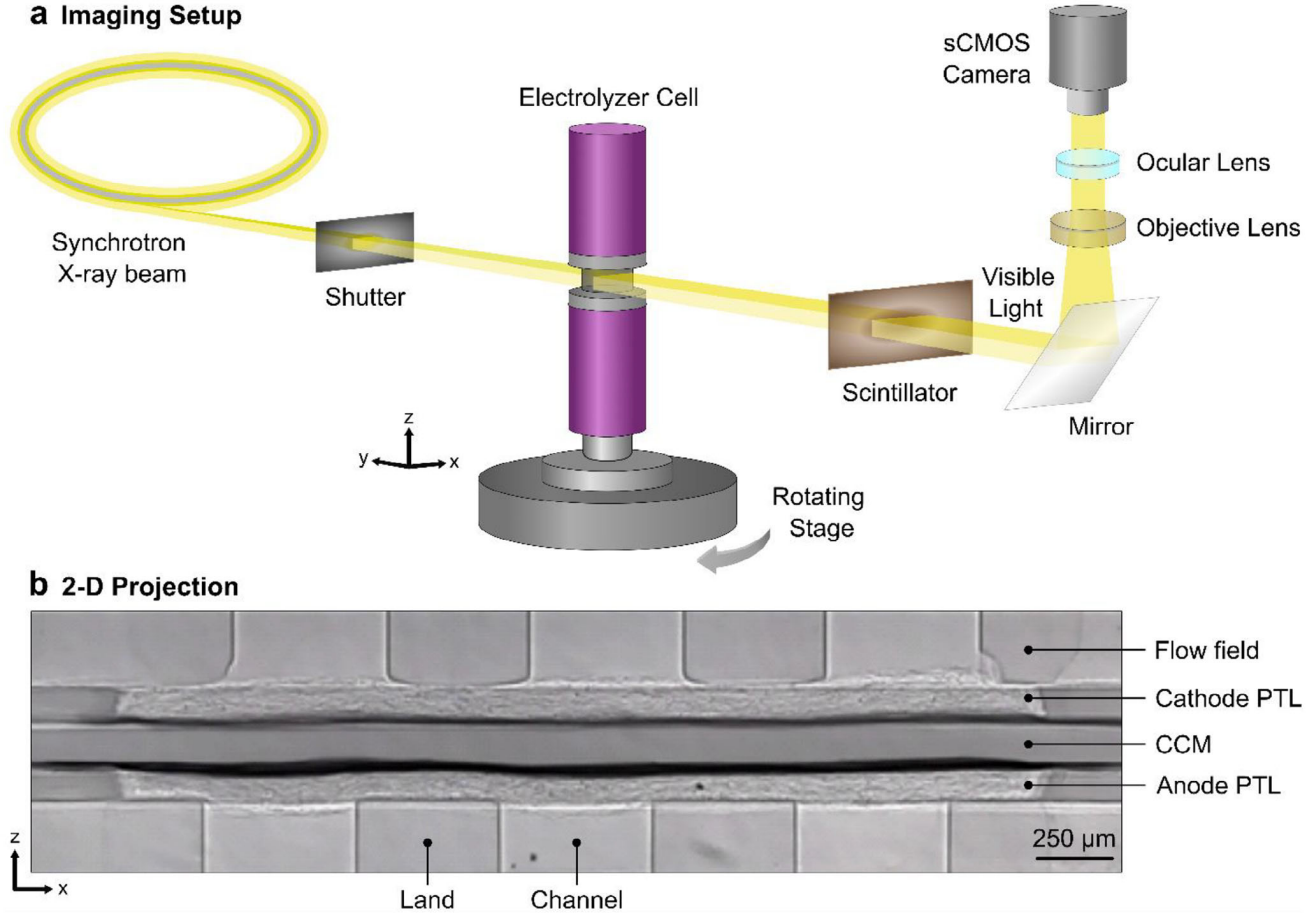
Schematic of operando X‐ray CT of the PEM electrolyzer at the 05B1‐1 BM beamline at CLS: a) The synchrotron X‐ray beam penetrates the PEM electrolyzer cell in the in‐plane orientation while the cell rotates on the rotating stage. b) Sample 2‐D projection image obtained of the PEM electrolyzer showing flow fields, anode and cathode PTLs, and the CCM.

To facilitate this investigation, a small‐scale yet representative PEM electrolyzer cell optimized for operando X‐ray CT is designed. A schematic of the experimental setup is shown in **Figure** **1**. 3‐D CT datasets are obtained and processed to reveal quantitative and statistical insights into gas bubble distributions in the PTL at varying operating temperatures. Quantitative 3‐D datasets are further correlated to electrochemical cell performance to reveal mechanistic links between operating temperature, oxygen removal, and bubble dynamics in the PTL. By directly connecting pore‐scale gas distributions to temperature‐dependent performance metrics, this work provides new mechanistic insights into how temperature influences oxygen removal and bubble dynamics in the PTL. These findings resolve ambiguities in the existing literature and represent a critical advance in the rational design of PEM electrolyzer systems.

## 2 Results and Discussion

We first present the operando 3‐D spatial distribution of gas bubbles in the carbon‐based anode PTL of the electrolyzer at each operating temperature, visualized with X‐ray CT (Section 2.1). We characterize and examine temperature‐dependent bubble size distributions (Section 2.2). Then, we examine the transport behavior of gas bubbles at the PTL/flow field (FF) interface and discuss the two‐phase flow in the channels (Section 2.3). Lastly, we present our electrochemical analysis to discuss the impact of temperature on electrolyzer performance with correlation to observed bubble distributions (Section 2.4).

### 2.1 Higher Temperature Leads to Lower PTL Bubble Volume and Saturation

**Figure 2:**
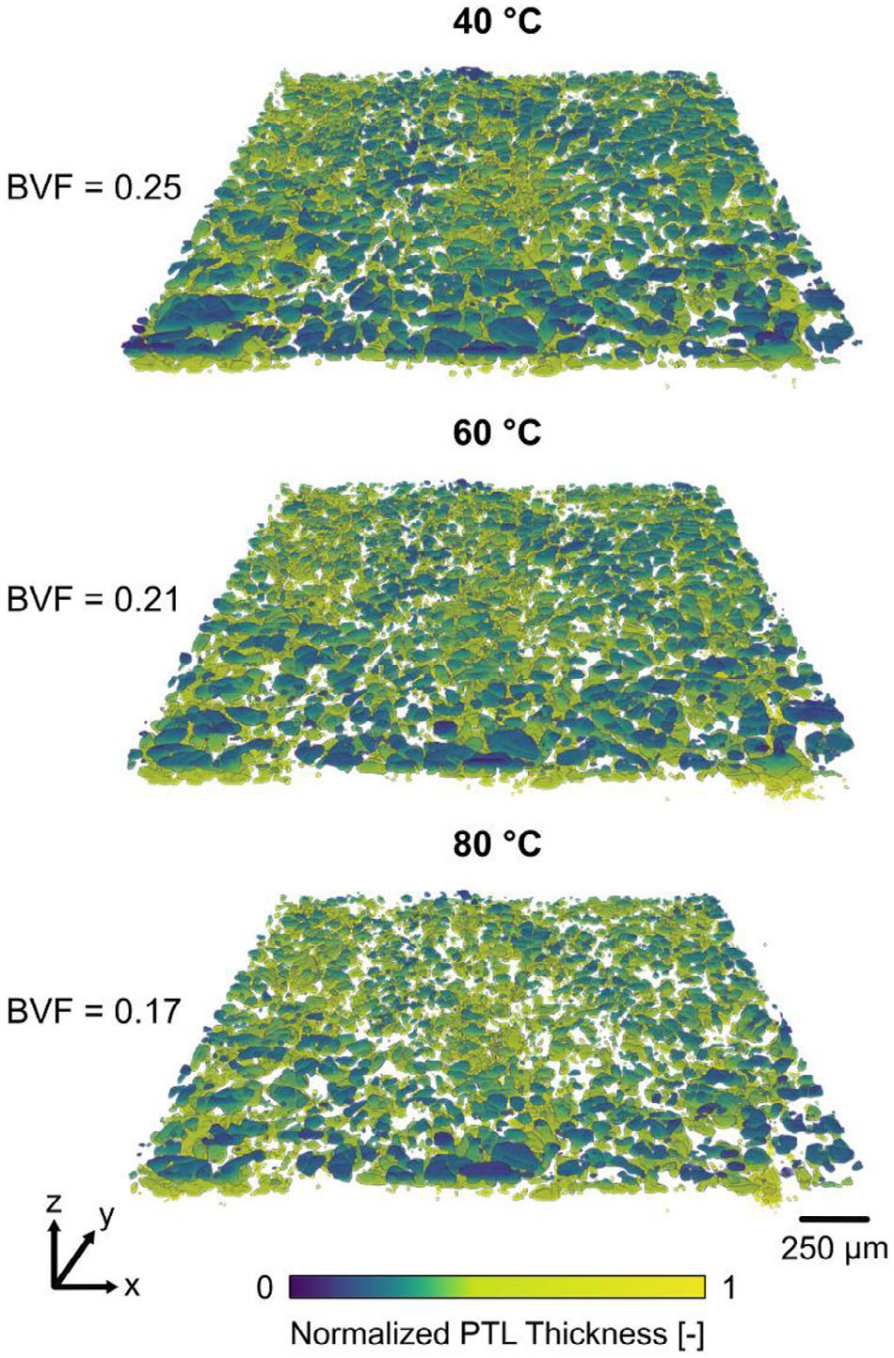
3‐D volume renderings of bubble distributions in the anode PTL at each operating temperature (40 °C, 60 °C, and 80 °C) at 4 A cm^−2^. The segmented gas distribution is color‐scaled along the normalized PTL thickness where 0 is the CL/PTL interface and 1 is the PTL/FF interface. Lower total gas bubble volume fraction (BVF) was observed at higher operating temperature.

The total gas bubble volumes in the anode PTL at operating temperatures of 40 °C, 60 °C, and 80 °C at a current density of 4 A cm^−2^ were determined via operando X‐ray CT (**Figure** **2**; detailed 3‐D rendering showing segmentation and bubble identification steps are shown in Video S1, Supporting Information). The total bubble volume fraction (BVF) within the PTL (excluding bubbles in the channels) was 0.25, 0.21, and 0.17 for temperatures 40 °C, 60 °C, and 80 °C, respectively (Figure 2). Notably, the total bubble volume was 32% lower at 80 °C compared to 40 °C.

We propose that temperature‐sensitive fluid parameters, which collectively reduce the capillary pressures required to drive two‐phase flow in the PTL, led to the lower gas bubble volumes at the higher temperatures (60 °C and 80 °C). The Young‐Laplace equation, which describes the two‐phase flow involving liquid water and gas in the PTL, is defined as:^[^ ^35^ ^]^

(1) $$P_{c}=\frac{2\gamma \cos\theta }{r}$$

where *P_c_* [Pa] is the capillary pressure, γ [N m^−1^] is the surface tension at the water‐gas interface, θ [^○^] is the contact angle of liquid water interfacing the solid surface, and *r* [m] is the effective pore radius. Both the surface tension, which is the interfacial tension between gas and liquid water, and the contact angle, which is related to the surface energy of the liquid water and solid surface, decreases with increasing temperature due to the rise of kinetic energy of water molecules, reducing the relative influence of intermolecular forces.^[^ ^54^ ^]^ This decrease in surface tension and contact angle at higher temperatures reduces the capillary pressure required for gas to displace water, aiding in the removal of gas bubbles from the PTL. In addition, the increase in local gas pressure with temperature leads to a greater pressure difference across the liquid‐gas interface, thereby more effectively overcoming capillary forces and promoting bubble removal. Moreover, the viscosity of liquid water is lower at higher temperatures, which reduces water transport resistance. The combined effect of these factors—lower surface tension, lower contact angle, increased gas pressure and lower water viscosity—synergistically enhance the displacement of gas bubbles away from and liquid water delivery to the catalyst layer at higher operating temperatures.

**Figure 3:**
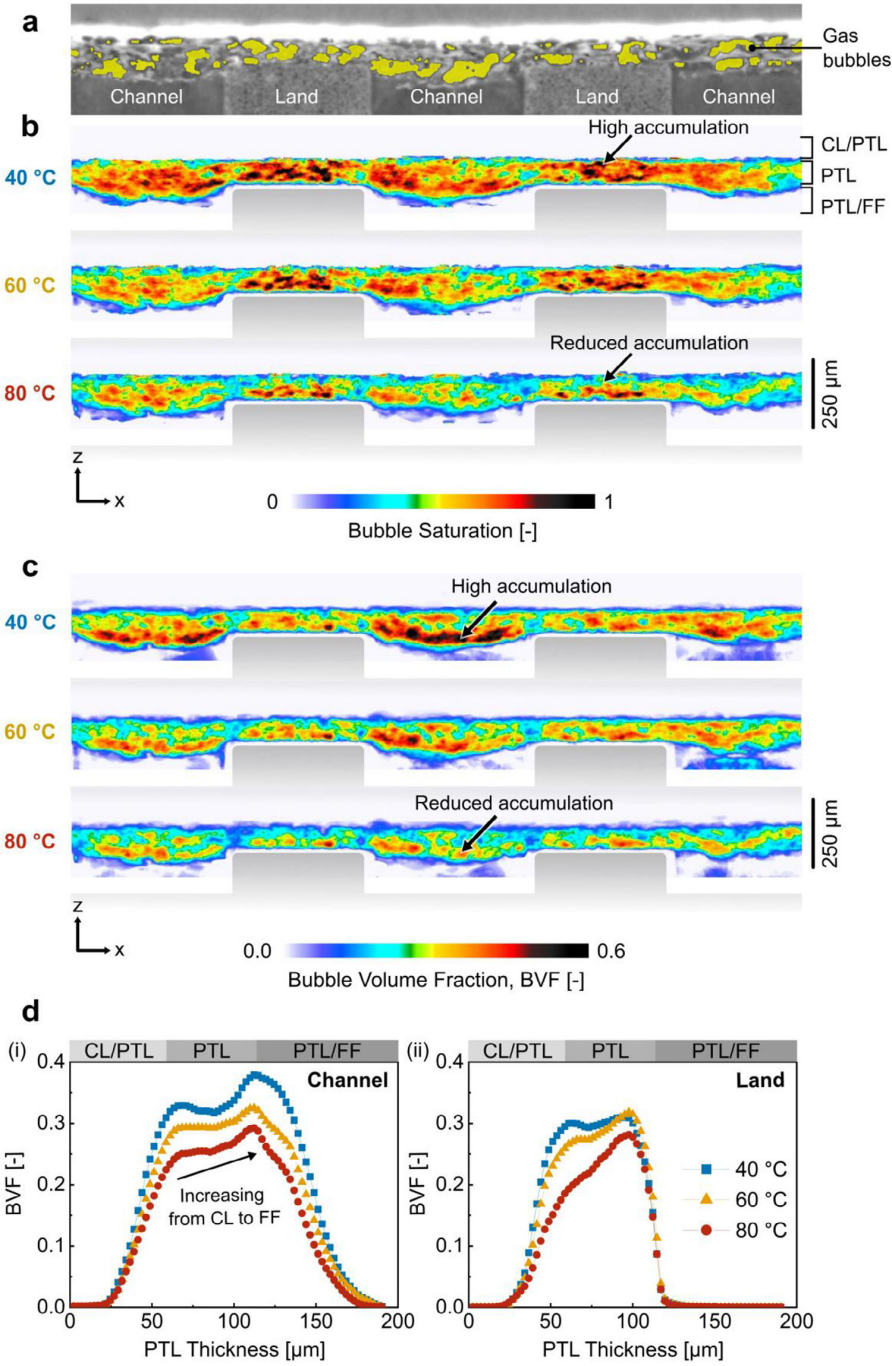
Spatial distribution of bubbles in the PTL for operating temperatures 40 °C, 60 °C, and 80 °C at a current density of 4 A cm^−2^. a) The relevant region of interest of the MEA shown by a gray scale planar image with segmented gas overlaid (yellow). b) Corresponding 2‐D spatial distributions of bubble saturation (summation along y‐direction) for each operating temperature. At increasingly higher operating temperatures, bubble saturation was consistently lower across the entire width (x‐direction) and thickness (z‐direction) of the PTL. c). Corresponding 2‐D spatial distributions of bubble volume fraction for each operating temperature. Regions of high bubble saturation and high bubble volume fraction differ due to the non‐uniform porosity of the PTL. d) Through‐plane profiles of bubble volume fraction (along the thickness of the PTL from the CL to the FF) for channel regions (left) and land regions (right) for each operating temperature.

While the total bubble volume fraction provides the bulk amount of gas accumulated within the PTL, bubble saturation shows the extent to which the accumulated gas occupies the available pore space within the PTL. To examine the trends in bubble saturation with temperature, we compared the in‐plane 2‐D spatial distributions of bubble saturation at 40 °C, 60 °C, and 80 °C in the region of interest shown in **Figure** **3**a. At increasingly higher operating temperatures (60 °C and 80 °C), bubble saturation was consistently lower across the entire width (*x*‐direction) and thickness (z‐direction) of the PTL (Figure 3b). The reduced bubble saturation at 80 °C compared to 40 °C was most pronounced under the land regions near the CL/PTL interface (noted by arrows in Figure 3b), representing a faster rate of bubble evacuation toward the channels (for subsequent removal from the PTL) at higher temperatures. Under the land regions near the PTL/FF interface, the reductions in bubble saturation were smaller since these bubbles were physically obstructed by the lands. Under the channel regions, there were greater reductions in bubble saturation near the PTL/FF interface than near the CL/PTL interface, indicating more effective bubble evacuation to the channels at higher temperatures. Overall, operating at higher temperatures (60 °C and 80 °C) resulted in improved bubble transport through the PTL, with the removal of bubbles away from the CL/PTL interface for subsequent removal via the channel regions, mitigating bubble accumulation not only under the channel regions but also under the land regions.

We note here that the higher bubble saturation under the land regions compared to the channel regions (Figure 3b) does not equate to more bubbles under the lands. When considering bubble volume fraction instead, which is shown as 2‐D distributions in Figure 3c, the bubble volume under the lands is lower than under the channels. This discrepancy between the regions of high bubble saturation from the regions of high bubble volume fraction is due to the non‐uniform PTL porosity that is considered in calculating bubble saturation. While the porosity of an uncompressed PTL is relatively uniform, the compressed carbon PTL experienced non‐uniform deformation due to compression against the flow field (illustrated by the bowing of the carbon PTL in Figure 3a). This uneven compression resulted in relatively larger pores under the channel regions—particularly at the PTL/FF channel boundaries—and smaller pores under the land regions. Hence, the bubble saturation under the lands (Figure 3b) is higher due to the smaller pore sizes (from compression). It is important to analyze both the bubble saturation and bubble volume fraction; bubble saturation allows us to isolate the impact of temperature on bubble transport, while bubble volume fraction allows us to isolate the impact of porosity. The 2‐D spatial distribution of gas bubbles in the orientation along the flow channels (from the inlet to outlet regions of the PTL) also shows reduced bubble volume fraction at higher temperatures (provided as supplementary data in Figure S1, Supporting Information (Appendix A)).

It is interesting to note that in our previous operando X‐ray CT work on PEM electrolyzers using titanium PTLs,^[^ ^55^ ^]^ we revealed accelerated membrane thinning during electrochemical operation which was attributed to the combined effects of compressive stress and membrane dehydration induced by gas accumulation near the CL/PTL interface. In this current work, the use of carbon PTLs on both the anode and cathode instead of titanium PTLs reduced the local compressive stress experienced by the membrane, since the lower stiffness of the carbon PTL allowed a portion of the compressive pressure to be absorbed through PTL deformation, rather than fully transferred to the membrane. This deformation in the carbon PTL resulted in material bowing (see Figure 3a) and created a through‐plane pore size gradient (with larger pores near the PTL/FF channel boundaries) effectively facilitating the removal of gas bubbles away from the CL/PTL interface, as shown by the ascending bubble volume profile from the CL/PTL to the PTL/FF interfaces (Figure 3d). Consequently, the effective mass transport of gas bubbles away from the CL/PTL interface supported adequate membrane hydration and thus, the membrane in the current cell configuration with carbon‐based PTLs experienced minimal operation‐induced deformation.

### 2.2 Higher Temperature Leads to Smaller Bubble Diameter in the PTL

**Figure 4:**
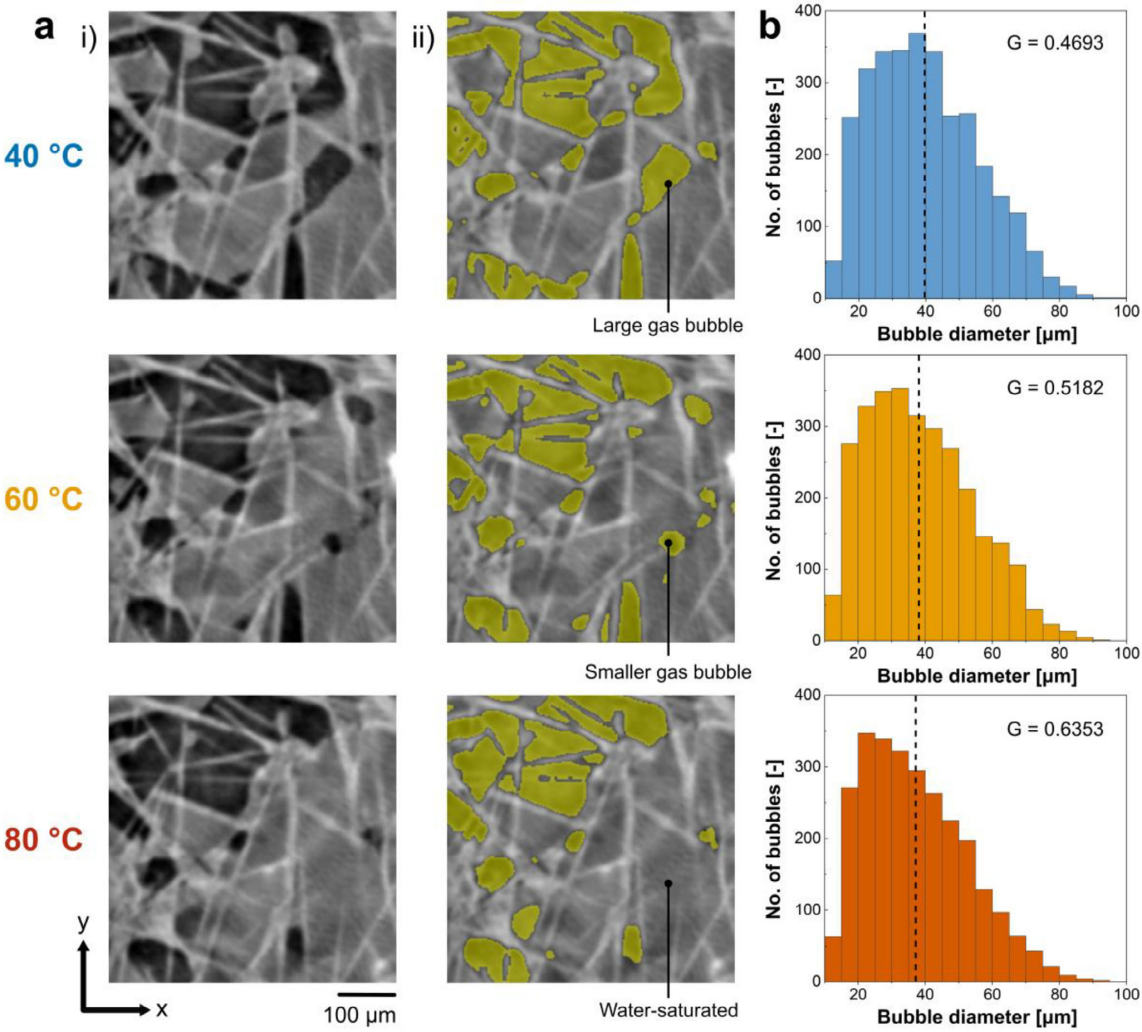
Bubble diameter distributions at each operating temperature: a) Internal x‐z planar views of pore‐scale gas distributions at identical locations of the PTL (i) are shown for temperatures 40 °C, 60 °C, and 80 °C at current density of 4 A cm^−2^ with (ii) the segmented gas in yellow overlaid onto the images. Certain voids which are filled with gas bubbles at lower temperature are saturated with water at higher temperatures. b) Bubble diameter distributions of pore networks extracted from the segmented gas distributions for temperatures 40 °C, 60 °C, and 80 °C at current density of 4 A cm^−2^. The mean bubble diameter is denoted by a dashed line on each histogram. The bubble size distribution at 80 °C compared to 40 °C presents a more right‐skewed distribution with a 35% increase in the Fisher‐Pearson coefficient of skewness, suggesting a greater occurrence of smaller bubbles at higher operating temperature.

To investigate the effects of operating temperature on bubble diameter in the PTL, the bubble size distribution was obtained for each operating temperature using the marker‐based watershed segmentation method.^[^ ^56^ ^]^ The bubble size distributions at 40 °C, 60 °C, and 80 °C are shown in **Figure** **4**b and their statistics are summarized in **Table** **1**. The total number of bubbles was 3100 and 2692 at 40 °C and 80 °C respectively, equaling to 13.2% less bubbles at 80 °C compared to 40 °C (Table 1). This reduction in bubble count is also qualitatively visible in Figure 4a, where some pores that were predominantly occupied by gas bubbles at 40 °C were increasingly occupied by water at 60 °C and 80 °C.

**Table 1:** Bubble diameter and pore diameter distributions.

Operating temperature [°C]	Mean bubble diameter [µm]	Median bubble diameter [µm]	Total bubble count [‐]	Bubble‐filled pores [%]	Fisher‐Pearson coefficient of skewness, G [‐]
40	39.8	38.4	3100	68.6	0.469
60	38.4	36.5	2938	65.0	0.518
80	37.2	35.1	2692	59.6	0.635
	**Mean pore diameter [µm]**	**Median pore diameter [µm]**	**Total pore count [‐]**		
Dry	42.5	40.0	4518		

To assess the statistical similarity of the bubble size distributions at each operating temperature, the two‐sample one‐sided K‐S test was applied to each combination pair of bubble size distributions. In all tests, the resulting p‐value was below the rejection threshold meaning that there exists a significant trend with changing temperature (**Table** **2**); that is, bubble sizes at 80 °C tended to be smaller than that at 60 °C which tended to be smaller than that at 40 °C. To quantify this trend, the Fisher‐Pearson coefficient of skewness was computed for each distribution (Figure 4a and Table 1). The skewness was higher at 80 °C with a value of 0.635, compared to a value of 0.469 at 40 °C, showing a stronger right‐tailed skew and a greater occurrence of smaller bubbles at higher temperatures. This temperature‐dependence of bubble diameter is particularly noteworthy, since if temperature only enhanced liquid water transport, we would expect a reduction in the total number of bubbles, but with a similar shape in bubble diameter distribution across the operating temperatures. Alongside the decrease in the mean and median bubble sizes (Table 1), the significant increase in skewness of bubble size distribution suggests smaller bubbles exist at higher temperatures, a correlation which has not yet been comprehensively discussed in the literature.

**Table 2:** K‐S test results for bubble diameter distributions.

Sample 1	Sample 2	Rejection threshold^a^	p‐value
40 °C	60 °C	0.0230	0.0015
40 °C	80 °C	0.0358	< 0.0001
60 °C	80 °C	0.0362	0.0119

^a)^ The rejection threshold was computed for α = 0.05, and the null hypothesis was rejected when the p‐value was less than the rejection threshold.

We attribute the higher number of smaller bubbles in the PTL at higher operating temperatures (60 °C and 80 °C) (Figure 4b) to changes in temperature‐sensitive fluid parameters affecting bubble nucleation which include solubility, diffusivity, and surface tension. The solubility of oxygen in liquid water decreases with increasing temperature, thereby lowering the supersaturation threshold and consequently promoting bubble nucleation at the electrode surface. Higher temperatures also lead to increased gas diffusivity, leading to a more rapid migration of gas molecules to nucleation sites.^[^ ^57^, ^58^ ^]^ Additionally, surface tension decreases with temperature, reducing the critical radius^[^ ^59^, ^60^ ^]^ for bubble nucleation and thus increasing the rate of nucleation. Lower surface tension at higher temperatures also leads to a lower bubble adhesive force, thereby decreasing the size threshold for detachment. The combination of faster nucleation rate and lower detachment size threshold contribute to a greater occurrence of smaller bubbles at higher temperatures. Furthermore, we propose that smaller bubbles encounter fewer physical obstructions as they migrate through the PTL, contributing to the lower bubble volume fraction observed at higher temperatures, as discussed in Section 3.1.

### 2.3 Higher Temperature Enhances Gas Removal and Breakthrough into Channels

**Figure 5:**
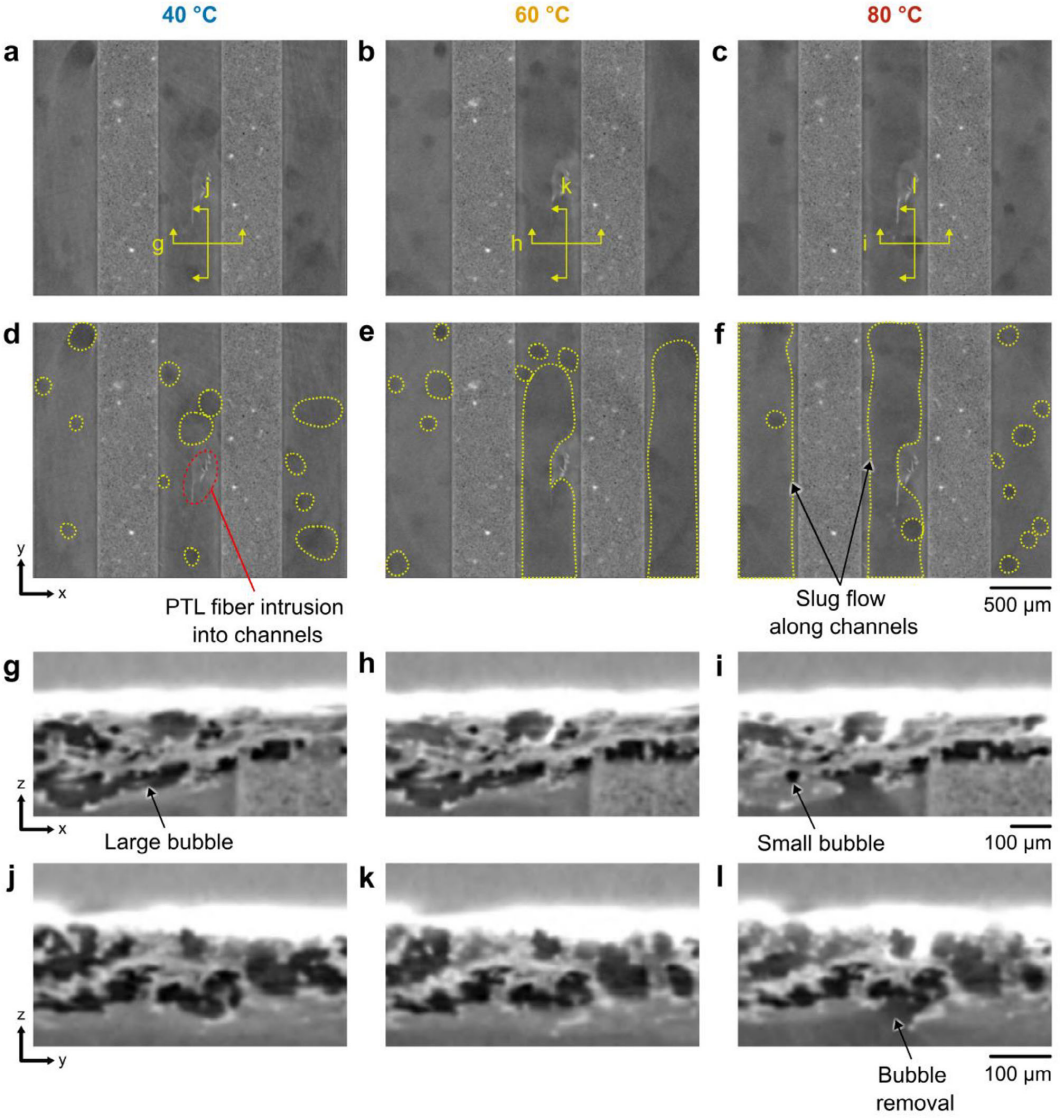
Gas transport along channels after breakthrough at the PTL/FF interface: a–c) Internal x‐y planar gray scale slices at the PTL/FF interface are shown with corresponding annotated images shown below d‐f) with identified bubbles and slugs for operating temperatures 40 °C, 60 °C, and 80 °C at current density of 4 A cm^−2^. Darker columnar regions along the channels suggests the movement of large slugs. g–i) Internal x‐z planar views of identical regions of the PTL and PTL/FF interface with cross sections denoted by horizontal yellow lines in (a–c). j–l) Internal y‐z planar views of identical locations at the PTL/FF interface with cross sections denoted by vertical yellow lines in (a–c). An example of a breakthrough gas bubble at a boundary pore (at the PTL/FF interface) is observed at 80 °C.

To elucidate the reduction in bubble volume in the PTL at higher temperatures, we examined the gas transport behavior in the anode flow channels near the PTL/FF interface (**Figure** **5**a–c). Each tomographic scan required 10 s to acquire and consisted of 500 projection images averaged into a single reconstructed volume. Consequently, the images presented here represent a time‐averaged snapshot of the liquid‐gas distribution during this 10 s interval rather than an instantaneous frame. In these images, bright regions correspond to water‐filled areas, dark regions correspond to gas‐filled areas, and intermediate grey values reflect regions intermittently occupied by gas and water (i.e., moving bubbles). Thus, the darkest regions indicate stagnant or long‐residence‐time bubbles, while semi‐dark regions suggest intermittent bubble passage. From a qualitative analysis of the internal planar view at the PTL/FF interface, we repeatedly identified columnar, semi‐dark regions along the channels at 60 °C and 80 °C (Figure 5b,c,e,f) that were much less apparent at 40 °C. These columnar regions at 60 °C and 80 °C imply the movement of large bubbles or slugs spanning the width of the channels that repeatedly pass through the channels. This behavior is attributed to a more rapid breakthrough of gas bubbles from the PTL at higher operating temperatures, which allows bubbles to coalesce into slugs in the flow field channels. To observe gas bubble breakthrough at the PTL/FF interface, internal cross sectional views of identical locations across temperatures are shown in Figure 5g–l. Given the 10 s acquisition window, the location of any individual bubble may be time‐dependent rather than temperature‐dependent; nevertheless, we highlight a representative bubble breakthrough behavior observed at 80 °C in Figure 5l. Previously, Lee et al.^[^ ^37^ ^]^ employed 2‐D through‐plane radiography to identify thicker layers of gas in the channels at higher temperature, which were hypothesized to result from an increased bubble detachment frequency. Our operando 3‐D imaging results provide direct evidence supporting this hypothesis and adds to the understanding of temperature‐dependent two‐phase flow in PEM electrolyzers.

### 2.4 Higher Temperature Leads to Improved Overall Cell Performance

**Figure 6:**
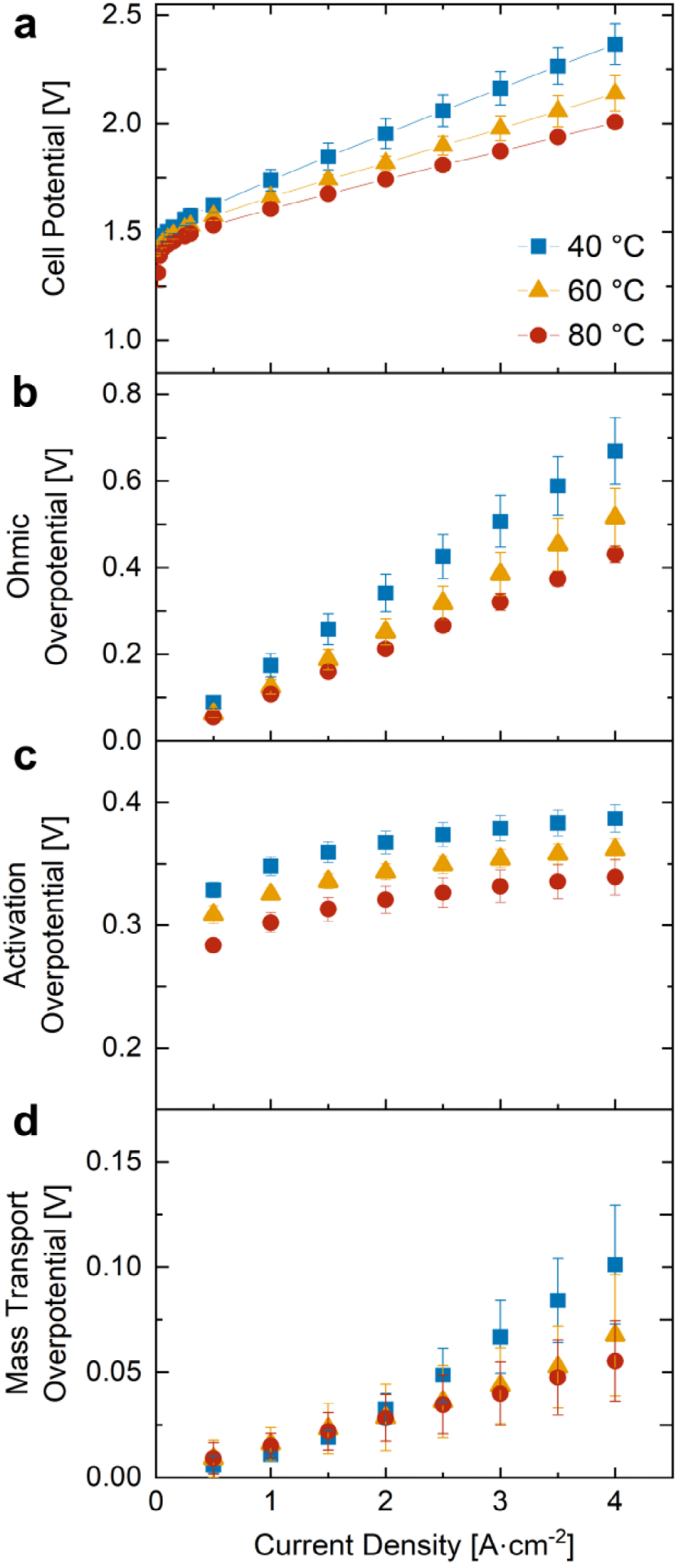
Temperature‐dependent Electrochemical Performance: a) polarization curves at the three operating temperatures (40 °C, 60 °C, and 80 °C) averaged from 6 distinct builds including the operando imaging experiment. Cell performance was improved at elevated operating temperatures for all current densities. b) Activation, ohmic, and mass transport overpotential all generally decreased at higher operating temperature. Error bars represent the standard deviation of the average of 6 experiments with separate cell builds (1 operando imaging experiment and 5 repeated in‐lab tests). Tafel plots are shown in supplementary data in Figure S4 (Supporting Information).

The performance of the electrolyzer cell was characterized via polarization curve measurements at 40 °C, 60 °C, and 80 °C, as shown in **Figure** **6**a. Cell performance improved with higher operating temperatures across all tested current densities, which is in agreement with previous literature.^[^ ^35^, ^36^, ^37^, ^38^, ^61^, ^62^, ^63^ ^]^ By calculating the respective losses (Figure 6b–d), it was found that the primary contributor to the improved performance at 80 °C compared to 60 °C and 40 °C was a lower ohmic overpotential (Figure 6b). The lower ohmic overpotentials at the higher temperatures (60 °C and 80 °C) is due to the higher ionic conductivity of the membrane, resulting from both elevated temperatures and enhanced membrane hydration due to lower gas accumulation. The activation overpotentials were also lower at 60 °C and 80 °C (Figure 6c), which is explained by a lower activation barrier at higher temperatures. Notably, slightly lower mass transport overpotentials were observed at 60 °C and 80 °C from a current density of 3 A cm^−2^ onwards(Figure 6c), with a maximum reduction of 0.05 V between 40 °C and 80 °C at 4 A cm^−2^. The lower mass transport overpotentials at these higher temperatures corresponded with the lower volumes of gas accumulation in the PTL, as seen in the operando images at 4 A cm^−2^. However, the overall reduction in mass transport overpotential, particularly from 60 °C to 80 °C, was smaller than expected, despite notable reductions in bubble volume fraction. (The total bubble volume fraction in the PTL was 0.25, 0.21, and 0.17 for 40 °C, 60 °C, and 80 °C respectively.) This suggests that the observed changes in PTL bulk saturation at 4 A cm^−2^ may not have had a substantial impact on the water/gas transport near the CL/PTL interface to a degree that would influence the mass transport overpotential. As discussed earlier, an ascending through‐plane bubble volume profile was observed from the CL/PTL to the PTL/FF interface, with gas bubbles accumulating near the PTL/FF interface, away from the reaction site. Minimal gas bubble accumulation near the CL/PTL interface across all operating temperatures facilitated adequate water delivery to the CL, which explains the relatively small decreases in mass transport overpotentials at increasingly higher operating temperatures.

The present study advances the fundamental understanding of temperature‐dependent two‐phase flow in PEM electrolyzers by providing clear mechanistic evidence, which is a necessary step toward informing optimal electrolyzer design and operation. However, the long‐term implications for operational stability remain to be explored. Prolonged high‐temperature operation can accelerate degradation pathways such as membrane dehydration, catalyst layer instability, and PTL corrosion or oxidation leading to wettability loss (for carbon and titanium PTLs, respectively),^[^ ^64^ ^]^ all of which may alter transport behavior over time. Future studies should integrate long‐duration operando diagnostics to couple temperature‐dependent transport behavior with evolving material properties under realistic operating conditions. This could include combining X‐ray CT with complementary in situ techniques such as electrochemical impedance spectroscopy, gas chromatography analysis, or post‐mortem material characterization to track performance losses alongside structural changes. Such investigations would enable the development of predictive models linking temperature effects to component durability and support optimized thermal management strategies.

## 3 Conclusion

This study investigates the impact of operating temperature on gas bubble distribution and transport behavior in the carbon‐based anode PTL of a PEM electrolyzer. Operando X‐ray CT was employed to visualize a PEM electrolyzer during operating temperatures of 40 °C, 60 °C, and 80 °C at a current density of 4 A cm^−2^. We reveal that higher temperature operation (80 °C compared to 40 °C) led to a lower total gas bubble volume fraction in the PTL (0.25 to 0.17; 32% reduction), and fewer bubbles (3100 to 2692; 13% reduction), indicating improved bubble removal at higher temperatures. Additionally, a stronger right‐skew of bubble size distribution at 80 °C compared to 40 °C indicated a greater occurrence of smaller bubbles at higher temperatures. The improved bubble removal and increased number of smaller bubbles are attributed to two main factors: i) improved water transport and ii) increased nucleation rate of smaller bubbles ‐ both due to temperature‐sensitive parameters such as surface tension, liquid water viscosity, gas solubility, and gas diffusivity. These combined effects promoted faster gas bubble removal and detachment from the PTL, leading to bubble coalescence and slugs in the anode channels at higher temperatures. Concurrent electrochemical analysis also indicated improved performance at higher operating temperatures, primarily due to reduced ohmic overpotentials as well as reductions (albeit less pronounced) in activation and mass transport overpotentials.

The findings of this study elucidate the influence of operating temperature on gas bubble transport behavior in a PEM electrolyzer with the first 3‐D physical evidence provided with operando X‐ray CT. Operating at higher temperatures is favorable in reducing gas bubble accumulation in the PTL; however, accelerated bubble removal from the PTL influences the two‐phase flow regime in the flow channels. These findings offer a comprehensive understanding of temperature effects on the two‐phase flow in PEM electrolyzers, providing critical insights to realize optimal operational parameters for next generation PEM electrolyzers.

## 4 Experimental Section

The temperature‐dependence of two‐phase liquid water‐gas transport in the PEM electrolyzer was visualized using operando X‐ray CT with simultaneous electrochemical characterization. First, the hardware and materials used in the PEM electrolyzer are described. Then, the electrochemical testing protocol and analysis used to characterize the cell performance is presented, and the imaging procedure is outlined. Finally, the detailed image processing steps to quantify gas distributions and bubble diameter are described.

### Electrolyzer Cell Hardware and Materials

A custom single‐cell PEM electrolyzer was designed for in‐plane operando X‐ray CT imaging. The design employed custom rotary unions and electrical slip rings (manufactured by Senring Electronics Co., Limited) to enable continuous rotation while preserving pneumatic and electrical connections. The 5‐layer membrane electrode assembly (MEA) consisted of a commercial Nafion N115 catalyst coated membrane (CCM) (Ion Power) with catalyst loadings of 1.0 mg cm^−2^ of iridium oxide (IrOx) on the anode and 0.3 mg cm^−2^ platinum (Pt) on the cathode, each coated to the dimensions of the active area. The active surface area was 3.0 mm × 2.4 mm. To ensure sufficient X‐ray transmission for water/gas visualization and differentiation, carbon‐based fiber substrates (Toray 060 Untreated, thickness of 190 µm) were selected as the porous transport layers (PTLs) on both the anode and cathode sides. Rigid 127 µm‐thick polytetrafluoroethylene (PTFE) gaskets were used to compress the PTLs on both the anode and cathode sides (30% compression from original thickness) and ensure sufficient sealing. To investigate only the effects of operating temperature on gas bubble dynamics, the same MEA was used for all three operating temperatures. The MEA was placed between two graphite flow fields, each with three parallel channels (0.5 mm wide and 0.5 mm deep) separated by lands (0.5 mm wide). The flow‐field plates were positioned between two gold‐nickel‐coated copper current collectors, which conducted electric current between the flow‐fields and the external circuit. For internal temperature measurement, a thermocouple (Type T thermocouple, Omega Eng.) was inserted below the channels of the anode flow field, 2.5 mm away from the active area on both the anode and cathode sides.

### Electrochemical Testing and Analysis

The cell was imaged at three operating temperatures: 40 °C, 60 °C, and 80 °C, where electrochemical testing was conducted simultaneously to link electrochemical performance parameters with observed gas distributions in the PTL. Temperature control was managed using a water circulation bath (Fisherbrand Isotemp heated bath circulator, Fisher Scientific), which provided pre‐heated deionized water to simultaneously heat both the anode and cathode end plates and rotary unions. For reactant supply, deionized water at atmospheric pressure was supplied to both the anode and cathode compartments at a rate of 1.8 mL min^−1^ using a peristaltic pump (Masterflex L/S precision variable‐speed console drive, Cole‐Parmer). Pulse dampeners were placed downstream of the peristaltic pump to ensure a stabilized reactant flow to the electrolyzer. A liquid water purge at a flow rate of 10 mL min^−1^ was used to flood the cell for 1 h before each temperature set point to remove any residual gas bubbles. At each temperature set point, cell performance was characterized using a potentiostat (Reference 3000, Gamry Instruments) via polarization curve measurements, involving current densities ranging from 0.05 A·cm^−2^ (open circuit voltage) to 4.0 A cm^−2^. To capture the Tafel region, the current density was increased in 0.05 A cm^−2^ increments up to a current density of 0.3 A cm^−2^. Additionally, hybrid electrochemical impedance spectroscopy (EIS) measurements were acquired at current density steps from 0.5 to 4.0 A cm^−2^, in 0.5 A cm^−2^ increments, across a frequency range of 0.1 Hz to 10 kHz.

To determine the internal losses of the operating PEM electrolyzer, the total cell potential *E_cell_* was measured and decomposed into the following components:

(2) $$E_{\mathrm{cell}}=E_{\mathrm{rev}}+\eta_{\mathrm{ohm}}+\eta_{\mathrm{act}}+\eta_{\mathrm{mtx}}$$

where *E*_cell_ is the measured cell potential [V], *E*_rev_ is the reversible potential [V], η_ohm_ is the ohmic overpotential [V], η_act_ is the activation overpotential [V], and η_mtx_ is the mass transport overpotential [V]. The reversible potential was estimated as a function of operating temperature, T [K] using an empirical correlation reported by LeRoy et al. for water electrolysis:^[^ ^65^ ^]^

(3) $$\begin{array}{cc} E_{\mathrm{rev}} & =1.5184-1.5421\times 10^{-3}\cdot T\\ & \;+9.523\times 10^{-5}\cdot T\cdot \ln T+9.84\times 10^{-8}\cdot T^{2} \end{array}$$

The ohmic overpotential was determined based on the current density and the measured ohmic resistance using Ohm's law:

(4) $$\eta_{\mathrm{ohm}}=i\cdot R_{\mathrm{ohm}}$$

where *i* is the current density [A cm^−2^], and *R*_ohm_ is the ohmic resistance [Ω cm^2^]. In this study, *R*_ohm_ was calculated for all current densities using the high‐frequency resistance (HFR) value from EIS measurements. Then, the iR‐corrected polarization curve was determined by subtracting the ohmic overpotential from the cell potential. The iR‐corrected curve was used to determine the activation overpotential which is defined as:^[^ ^54^ ^]^

(5) $$\eta_{\mathrm{act}}=b\cdot \log_{10}\left(\frac{i}{i_{0}}\right)$$

where *b* is the Tafel slope [V dec^−1^], *i* is current density [A cm^−2^], and *i*_0_ is the apparent exchange current density [A cm^−2^]. The Tafel region of the iR‐corrected curve ranging from 0.03 to 0.3 A cm^−2^ was fitted to determine the Tafel slope and exchange current density (Tafel plots are shown in supplementary data in Figure S4, Supporting Information (Appendix A)).

### X‐Ray CT Data Acquisition

X‐ray tomographic image acquisition was conducted at Beamline 05B1‐1 at the Biomedical Imaging and Therapy (BMIT) facility at the Canadian Light Source (CLS).^[^ ^66^ ^]^ The electrolyzer cell was oriented in the in‐plane direction of the X‐ray beam (i.e., the membrane was parallel to the beam) (see Figure 1). Upstream of the sample, low‐energy photons were removed from the X‐ray white beam using a combination filter composed of 0.8 mm‐thick aluminum and 0.25 mm‐thick tin. After passing through the electrolyzer cell, the synchrotron X‐ray beam (filtered white beam) was absorbed by a 50 µm thick LuAG scintillator (CRYTUR spol. s r. o), which converts the X‐ray irradiance to visible light. The visible light was then relayed to a digital scientific complementary metal‐oxide‐semiconductor (sCMOS) camera (Andor Marana 4.2B‐11) with 4.5 × Magnification (Optique Peter, Lentilly, France). For a single CT scan, a total of 500 2‐D projections were taken along a 180‐degree rotation with an exposure time of 20 ms per projection, resulting in a total CT scan time of 10 s. A sample projection image showcasing the MEA layers is shown in Figure 1b. A pixel size of 2.44 µm was achieved, with a field‐of‐view (FOV) of 4.9 mm × 3.5 mm. The cell was imaged at each operating temperature during a current density of 4 A cm^−2^ once a stable voltage response was observed. After the operando imaging experiments, the cell was purged with ambient air at a flow rate of 10 mL min^−1^ using the peristaltic pump for 2 h before a dry scan was obtained to serve as a reference for the dry PTL structure.

### Image Reconstruction and Segmentation

**Figure 7:**
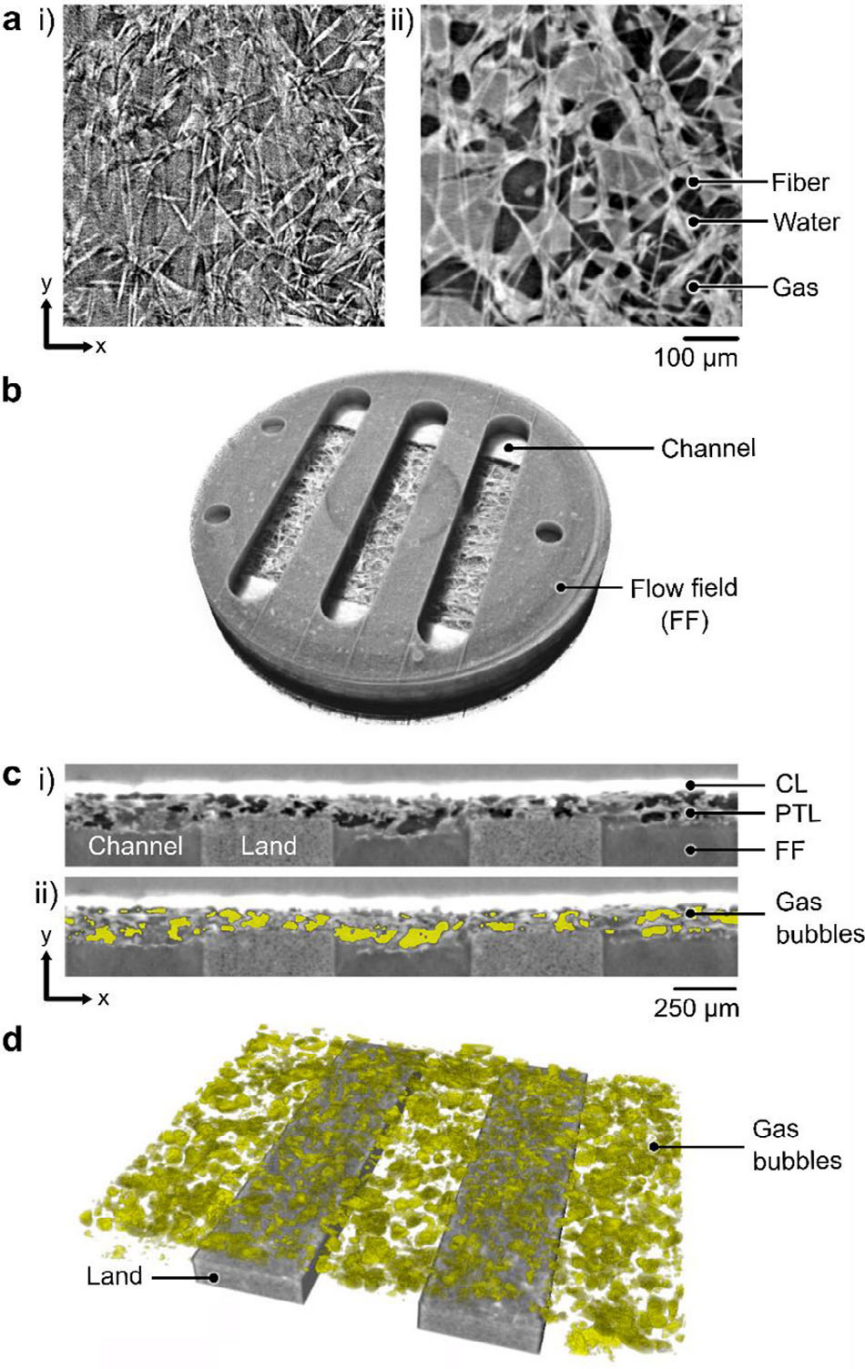
Image processing and segmentation of 3‐D gas distributions: a) A reconstructed slice (i) without and (ii) with phase retrieval. Phase retrieval reconstruction enhanced contrast between phases of fiber, water, and gas. b) 3‐D rendering of the single cell PEM electrolyzer housed between graphite FFs with three 0.5 mm wide channels. c) Gray scale threshold segmentation of gas bubbles in the cropped region of interest. The cropped gray scale image stack in (i) includes the CL, carbon PTL, and FF with three channels and two lands. The segmented gas distribution is overlaid in yellow onto the image stack in (ii). d) 3‐D volume rendering of segmented gas bubble distribution in the anode compartment overlaid onto anode FF with two 0.5 mm wide lands. Operando X‐ray CT successfully visualized pore‐scale gas bubbles.

The 2‐D radiographic projections of each tomography scan were reconstructed into 3‐D image stacks using ufo‐tofu software kit,^[^ ^67^ ^]^ an in‐house open‐source reconstruction graphical user interface at the CLS. Before reconstruction, a background correction was first applied by subtracting a dark‐field image (an average of 50 images acquired in the absence of the incident X‐ray beam) from each projection image to correct for the background noise of the camera. A normalization was also applied using a flat‐field image (an average of 500 images acquired in the absence of the sample) to correct for the inhomogeneities of the incident X‐ray beam, scintillator screen, and camera. Paganin's phase retrieval method,^[^ ^68^ ^]^ which was previously used for fuel cell liquid water visualization,^[^ ^40^ ^]^ was also applied prior to reconstruction to improve the contrast between water and gas phases in the pores of the PTLs, as illustrated in **Figure** **7**a. The key parameters as inputs for the Paganin algorithm were photon energy, pixel‐size, sample‐to‐detector distance, and delta‐to‐beta ratio (*δ/β*). The parameters *δ* and *β* represent the real and imaginary parts of the refractive index, n (n = 1 ‐ *δ* ‐ i*β*) for a homogenous material.^[^ ^68^ ^]^ For multi‐phase samples, *δ/β* could be tuned to optimize the contrast of the desired phase (gas in this case) in reconstructed images.^[^ ^40^, ^69^ ^]^ For images in this work, the energy was set to 27.0 keV, the sample‐to‐detector distance was 0.088 m, and *δ/β* was tuned to be 500. Finally, the filtered back projection algorithm^[^ ^67^ ^]^ was used to reconstruct the volume. The 3‐D rendering of the reconstructed single cell PEM electrolyzer is shown in Figure 7b (detailed 3‐D rendering showing segmentation and bubble identification steps are shown in Video S1, Supporting Information).

Prior to segmentation, the reconstructed image stacks were pre‐processed using Fiji^[^ ^70^ ^]^ to rotate, crop, and clip the grayscale histograms to the same range to minimize discrepancies between scans. Image registration and segmentation were then performed using DragonFly (Object Research Systems).^[^ ^71^ ^]^ Image registration was utilized to align each image stack to ensure that the same region of interest was consistently cropped for accurate analysis of gas phase distributions. This analysis focused on the anode side at the central region of the active area, encompassing three channel and two land regions (cropped domain was 2440 × 2000 × 300 µm^3^, as shown in Figure 7c,d). Since phase retrieval reconstruction produced sufficient contrast of the gas phase against the liquid/solid phases, a global manual threshold was implemented using the distinct gray scale value range for the gas phase.^[^ ^45^, ^52^, ^72^ ^]^ Next, a noise removal step involving a 3‐D erosion of 1 voxel length followed by a 3‐D dilation of 1 voxel length was applied.^[^ ^46^ ^]^ Similarly, the gray scale range of fiber was used to manually segment the void and solid phases of the PTL microstructure in the dry reference scan. The resulting 3‐D distribution of segmented gas is shown in Figure 7d.

Image analysis at open circuit voltage (OCV) revealed that not all voids of the PTL were fully saturated with water, indicating a non‐zero initial gas saturation. However, the measured initial gas saturation at the OCV state was similar for all temperature set points (40 °C, 60 °C, and 80 °C), and an increase in gas content was observed in the images starting at a current density of 50 mA cm^−2^, indicating the invasion of produced oxygen bubbles. Hence, in this study, gas phase distributions were reported on instead of oxygen content specifically.

### Bubble Volume Fraction and Saturation

To analyze the temperature‐dependence of gas distributions in the PTL, the gas bubble volume was quantified at each operating temperature. The bubble volume fraction, *BVF* [‐], is computed as:

(6) $$BVF=\frac{V_{g}}{V}$$

where *V*_g_ [µm^3^] is the volume of gas, defined as the number of voxels classified as gas phase multiplied by the voxel volume (2.44^3^ µm^3^), and *V* [µm^3^] is the total volume, defined as the number of voxels within the region of interest (a single image slice or volume) multiplied by the voxel volume. While total bubble volume fraction in the PTL is reported, the 2‐D spatial distribution of bubble volume fraction is also analyzed which is calculated by summing the total BVF in the y‐direction:

(7) $$BVF_{x,z}=\frac{\sum_{i=1}^{y}V_{g,i}}{V_{y}}$$

where Σ*V*_g,i_ [µm^3^] is the total volume of gas along y‐coordinates ranging from *i* = 1 *to* *i* = *y* at the specified x‐z coordinates, and *V_y_* [µm^3^] is the total volume, defined as the number of voxels in the y‐direction multiplied by the voxel volume (2.44^3^ µm^3^). Next, the gas bubble saturation *S_g_* [‐] in the PTL is computed at each operating temperature as:

(8) $$S_{g}=\frac{V_{g}}{V_{p\;}}$$

where *V*_p_ [µm^3^] is the PTL pore volume, defined as the number of voxels classified as pore space in the PTL (from the dry scan) multiplied by the voxel volume. Similarly, the 2‐D spatial distribution of bubble saturation is calculated as follows:

(9) $$S_{g,x,z}=\frac{\sum_{i=1}^{y}V_{g,i}}{V_{p,y}}$$

where *V*_*p*, *y*_ [µm^3^] is the total pore volume, defined as the number of voxels classified as pore space in the PTL in the y‐direction multiplied by the voxel volume.

### Bubble Size Distribution

The bubble size distribution was calculated from the segmented gas phase volume using the pore network extraction function in OpenPNM (opensource pore network modeling software package)^[^ ^73^ ^]^ plug‐in within DragonFly. A representative pore network was extracted from the 3‐D gas volume using the marker‐based watershed segmentation method,^[^ ^56^ ^]^ where the gas bubbles were represented as pores in the pore network. The bubble diameter was determined by calculating the diameter of a sphere with the equivalent volume as the identified bubble. The same pore network extraction was performed for the segmented dry PTL microstructure to acquire the number of total pores in a dry PTL. The ratio of bubbles from operando scans to total pores from the dry scan were then used to quantify the percentage of bubble‐filled pores for each operating temperature.

To assess the statistical similarity between the bubble size distributions from the three operating temperatures, the two‐sample one‐sided Kolmogorov‐Smirnov (K‐S) test was applied on every combination pair of bubble size distributions.^[^ ^74^ ^]^ Using a significance value of 0.05, the p‐values from each test were compared against the rejection threshold for the null hypothesis that one distribution does not contain significantly larger bubble sizes than the other. Instances where the null hypothesis was rejected would indicate both a significant difference between the distributions as well as identify a correlation of bubble size with operating temperature.

Next, to evaluate the change in shape of the bubble size distribution with changing operating temperature, the skewness of each distribution was computed. Skewness measures the symmetry of a distribution, where negative skewness indicates a greater occurrence of larger values, and positive skewness indicates a greater occurrence of smaller values. Therefore, in this study, the Fisher‐Pearson coefficient of skewness^[^ ^75^ ^]^ was used to compare skewness and provide insight into the proportion of bubbles sizes at each operating temperature. The Fisher‐Pearson coefficient of skewness, G [‐], is defined as:

(10) $$G=\frac{n}{\left(n-1\right)\left(n-2\right)}\sum_{i=1}^{n}{\left(\frac{x_{i}-\bar{x}}{s}\right)}^{3}$$

where $\bar{x}$ [µm] is the average bubble size, *s* [µm] is the standard deviation for a given distribution, and *n* [‐] is the number of total bubbles for a given distribution, with $x_{i}$ [µm] representing each bubble size for i=1,2,3,…n.

## Conflict of Interest

The authors declare no conflict of interest.

## Author Contributions

C.T.H. performed conceptualization, methodology, formal Analysis, investigation, wrote the original draft, wrote, reviewed, and edited the draft, visualization, and project administration. P.S. performed conceptualization, methodology, investigation, and wrote, reviewed, and edited the draft. L.K. performed methodology, investigation, and wrote, reviewed, and edited the draft. S.G. and M.A.W. performed methodology, software, investigation, wrote, reviewed, edited the draft, and provided resources. A.B. performed conceptualization, methodology, supervision, project administration, funding acquisition, wrote, reviewed, and edited the draft, and provided resources.

## Supporting information



Supporting Information

Supplemental Video 1

## Data Availability

The data that support the findings of this study are available from the corresponding author upon reasonable request.
