# Supplementary material for: The Role of Operating Temperature on Pore‐Scale Gas Transport in Polymer Electrolyte Membrane Electrolyzers
Source: Adv Sci (Weinh). 2025 Sep 8;12(44):e07606. doi: 10.1002/advs.202507606 (PMC12667457; doi:10.1002/advs.202507606)
Supplement: Supplementary file 1 — Supporting Information [file ADVS-12-e07606-s001.docx]

# Supporting Information

**The Role of Operating Temperature on Pore-scale Gas Transport in Polymer Electrolyte Membrane Electrolyzers**

*Chaeyoung T. Ham, Pranay Shrestha, Leya Kober, Sergey Gasilov, M. Adam Webb, and Aimy Bazylak**

**
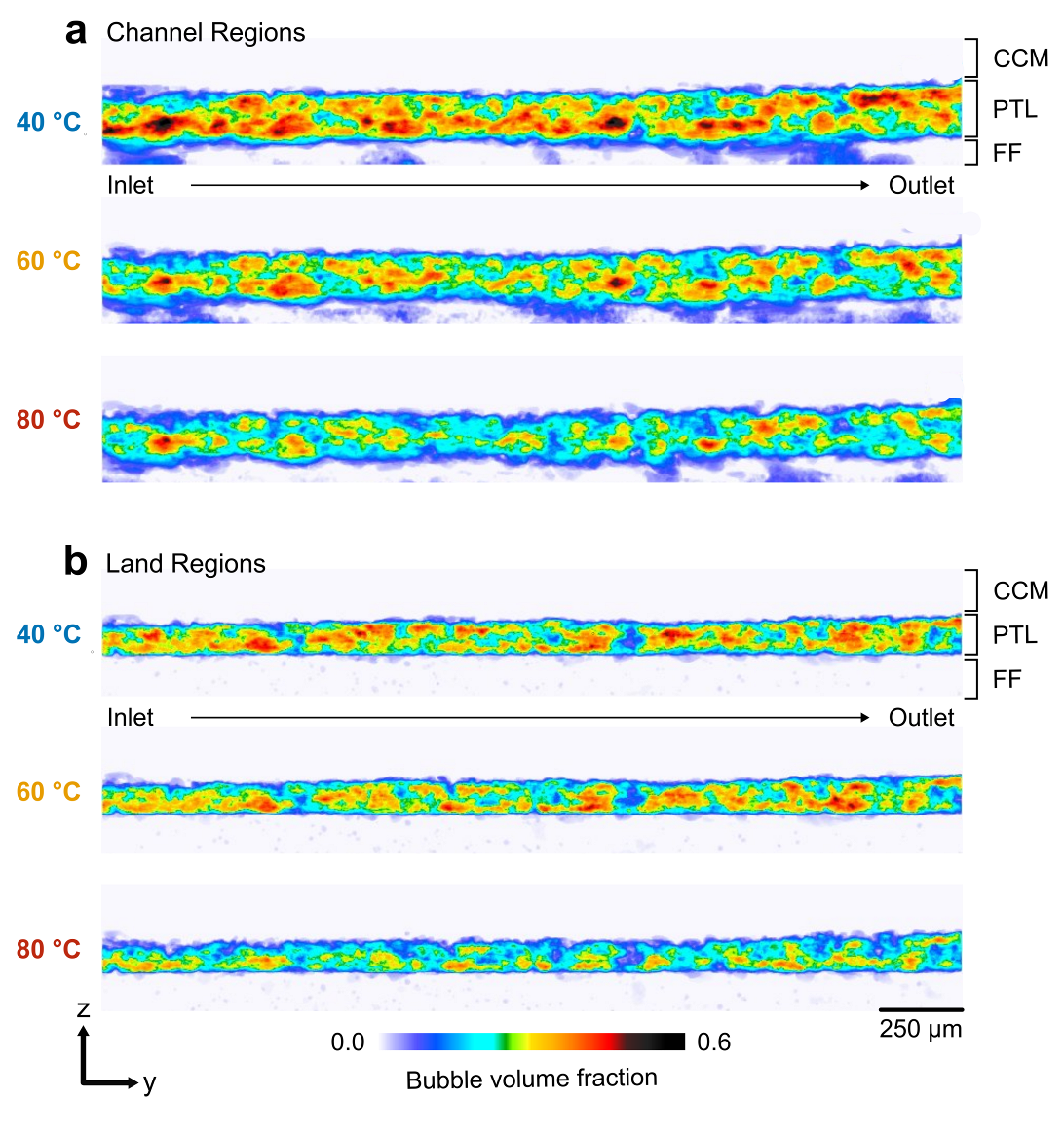
**

**Figure S1:** 2-D spatial distributions of bubble volume fraction in the PTL (along flow channels from inlet to outlet) for operating temperatures 40 °C, 60 °C, and 80 °C at a current density of 4 A cm^-2^. Corresponding 2-D projections of total bubble volume fraction (Z-projects along x-direction) at operating temperatures 40 °C, 60 °C, and 80 °C at current density of 4 A cm^-2^. Relatively homogeneous distribution of bubbles was observed from the inlet (left) to the outlet (right) of flow field channels.

At each operating temperature, we also examined the 2-D spatial distribution of bubble volume fraction along the orientation of the flow channels under land and channel regions of the PTL separately (Figure S1). At increasingly higher operating temperatures (60 °C and 80 °C), bubble volume fraction was consistently lower across the entire length (*y*-direction) and thickness (z-direction) of the PTL (Figure S1). Previously in Figure 3c, we compared the bubble volume under the land and channel regions, and the reduction in bubble volume fraction at higher temperatures was more pronounced under the channel regions. Along the direction of the flow channels (comparing the inlet region to the outlet region), the reduction in bubble volume at higher temperatures is homogeneous throughout the PTL (Figure S1).

^
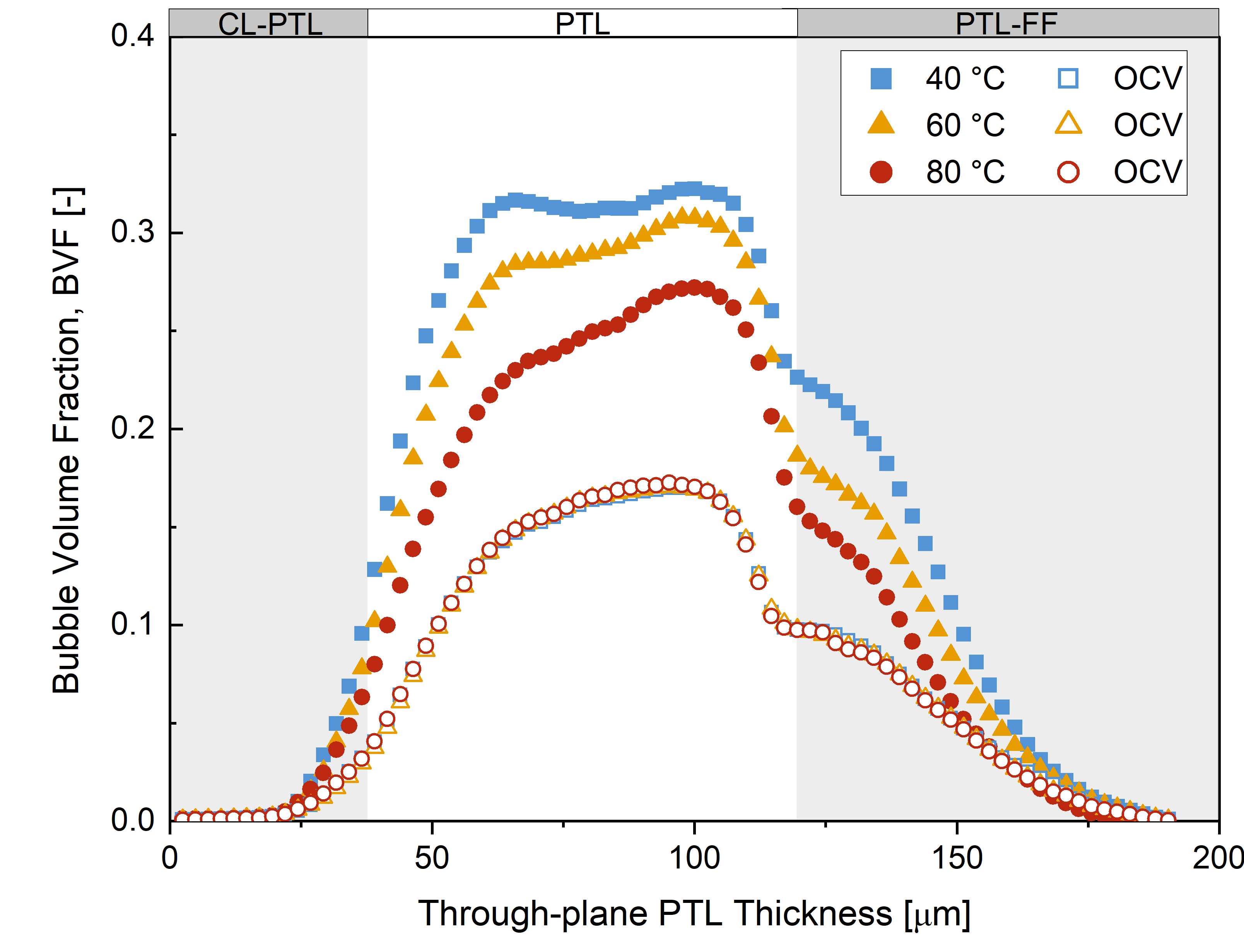
^

**Figure S2:** Gas content in the PTL (through-plane profiles along the thickness of the PTL from the CL to the FF) for operational scans at temperatures of 40 °C, 60 °C, and 80 °C at a current density of 4 A cm^-2^ and at open-circuit voltage (OCV) before each temperature operation. The consistent distribution of bubbles at OCV for each operating temperature represents consistent dry spots of the PTL when no gas is generated. The consistent gas content at OCV allows us to correlate the changes in gas content to the change in operating temperature.

In this study, we noted that all pores were not wetted at OCV, since untreated carbon PTLs can contain hydrophobic pores. To ensure transparency, we quantified the gas content at OCV (0 A cm⁻²) for all operating temperatures (see Figure S2). The consistent distribution and magnitude of gas content at OCV across all temperatures suggest these represent stable and repeatable dry spots rather than temperature-dependent differences. We also note that, in the operational scans (4 A cm⁻²), bubble production and accumulation were clearly observed, and distinct bubble distribution trends are seen between each temperature setpoint, which correlate to temperature-dependent fluid properties.


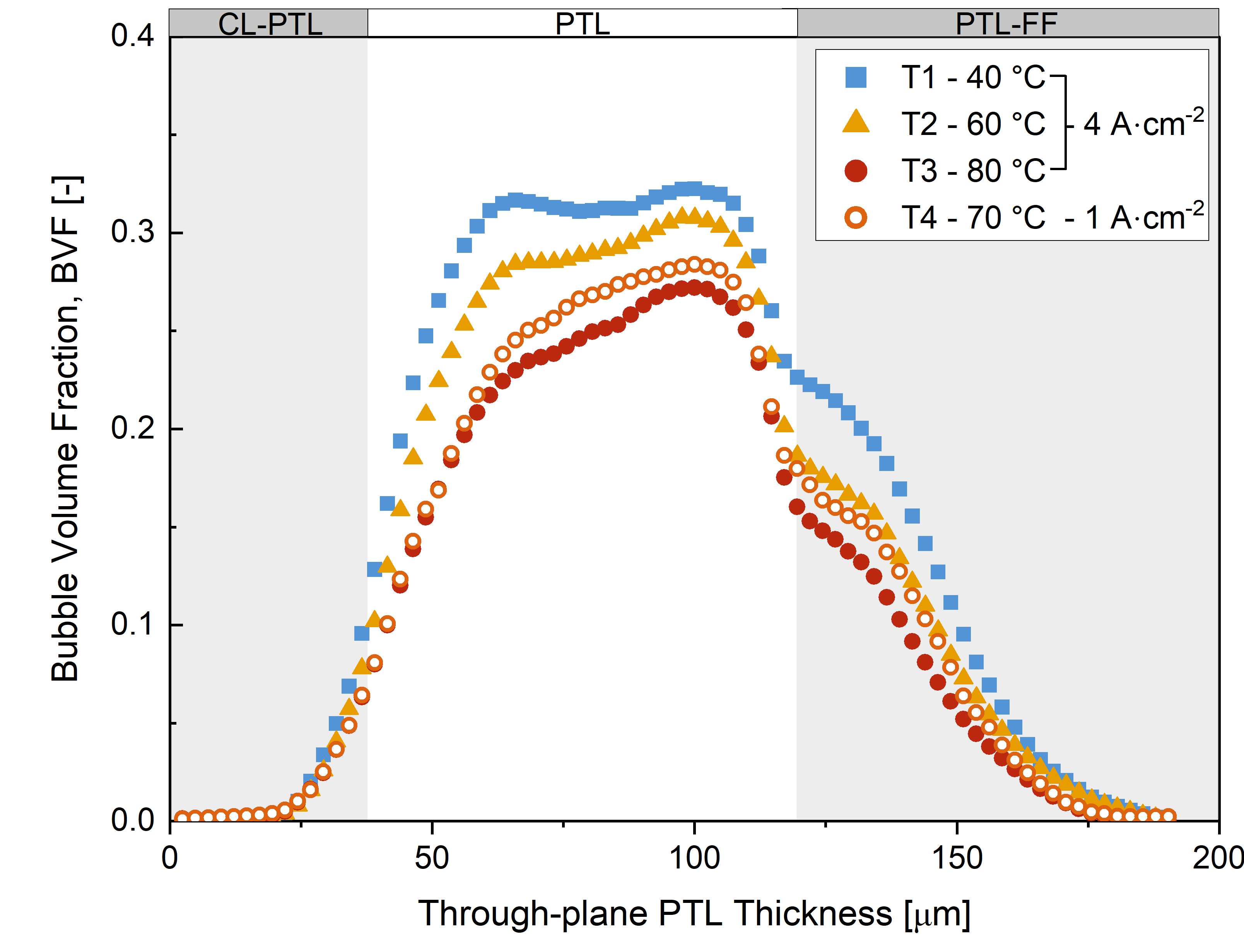


**Figure S3:** Gas content in the PTL (through-plane profiles along the thickness of the PTL from the CL to the FF) for an additional operational scan at a temperature of 70 °C conducted after the 40 to 80 °C sequence which was presented in our study; the observed increase in gas content at this later scan supports our conclusion that temperature, rather than operation time, was the dominant factor influencing gas content.

Previous studies have shown that untreated carbon PTLs can gradually become more hydrophilic due to carbon corrosion.^[76]^ In our experiments, we do not see any indication of change in wettability of the carbon GDL since the gas content and profile at OCV conditions for each operating temperature were both stable and repeatable (see Figure S2). To further verify that the changes in gas distributions in our experiments were temperature-induced and not time-dependent with carbon corrosion in play, we operated the cell at 70 °C after the 40 → 80 °C sequence; the observed increase in gas content at this later scan supports our conclusion that temperature, rather than operation time, was the dominant factor influencing gas content.

**
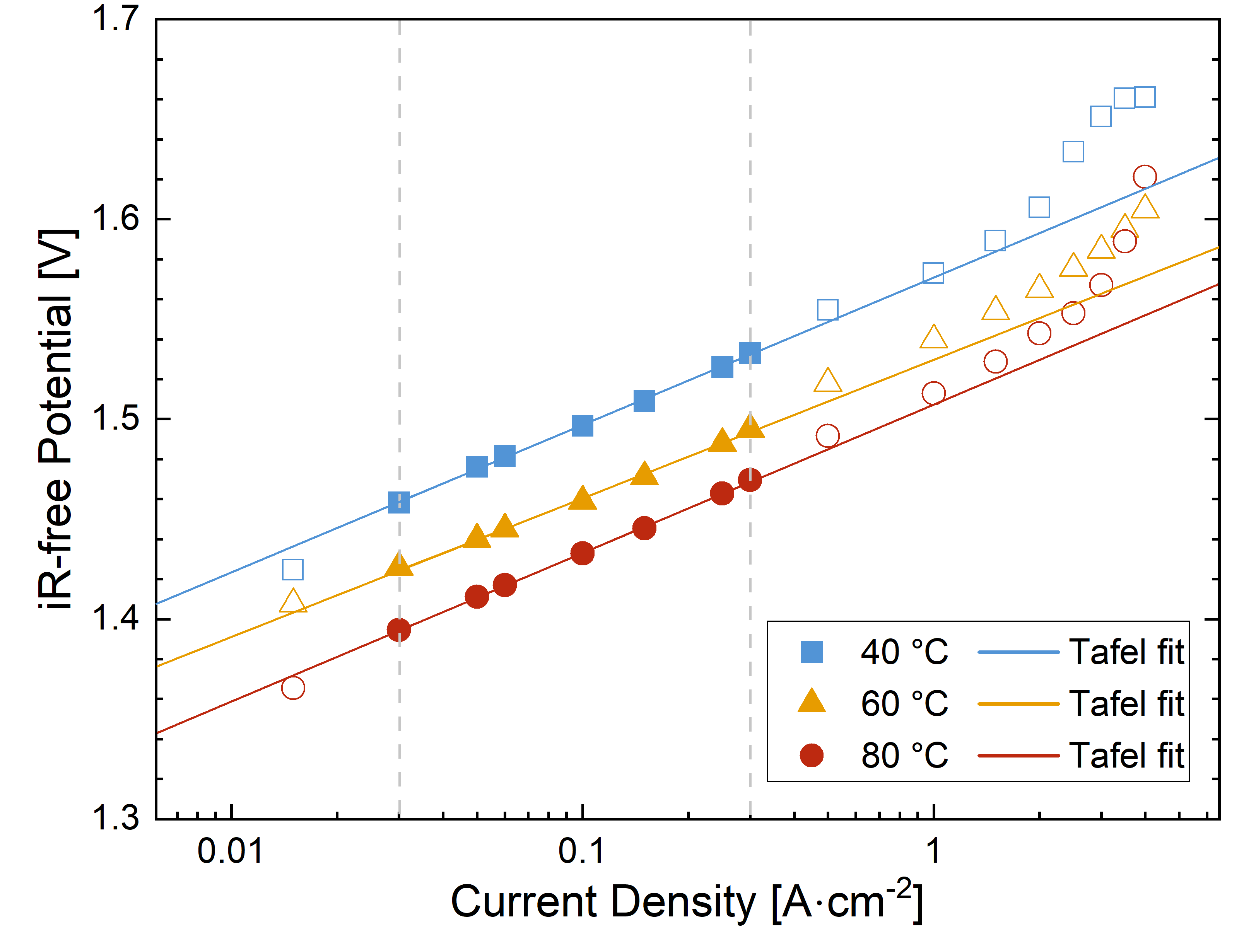
**

**Figure S4:** Tafel plots for determining the activation overpotential at each operating temperature. The Tafel region was considered from 30 mA cm-2 to 300 mA cm-2, indicated between dashed grey lines. This range was justified by the slight lift-off of the iR-corrected cell potential from the Tafel line when i > 0.3 A cm-2. The extracted Tafel slopes (68-74 mV dec-1) and high R2 values (>0.99) confirm that the parameters were obtained from a region where Tafel assumptions are valid.

**Supplemental Video:** 3-D Gas Segmentation and Bubble Identification
